# SERBP1 is required for efficient HR repair and cisplatin chemoresistance in lung adenocarcinoma

**DOI:** 10.1038/s41420-026-03017-x

**Published:** 2026-03-19

**Authors:** Yifei Xie, Qiongju Chen, Nana Tang, Yuanyuan Zeng, Jian Zhao, Yang Yang, Chang Li, Jianjun Li, Jianjie Zhu, Jian-an Huang, Zeyi Liu

**Affiliations:** 1 Department of Pulmonary and Critical Care Medicine, the First Affiliated Hospital of Soochow University, Suzhou, China; 2 Institute of Respiratory Diseases, Soochow University, Suzhou, China; 3 Suzhou Key Laboratory for Respiratory Diseases, Suzhou, China

**Keywords:** Tumour biomarkers, Prognostic markers

## Abstract

Resistance to cisplatin limits its clinical efficacy in LUAD patients and leads to poor prognosis. SERPINE1 mRNA binding protein 1 (SERBP1), an RNA-binding protein, is associated with tumorigenesis and progression. However, its specific role in cisplatin resistance and underlying mechanism in LUAD remain unclear. Here, we investigated the hypothesis that SERBP1 drives cisplatin resistance by reinforcing DNA damage repair capacity. We found that SERBP1 was consistently upregulated in cisplatin-resistant LUAD cells compared with cisplatin-sensitive counterparts. Gain-of-function and loss-of-function experiments demonstrated that SERBP1 promoted cisplatin resistance in LUAD. Mechanistically, SERBP1 contributes to cisplatin resistance by stabilizing BRCA1 mRNA, thus activating HR repair mediated by RAD51. Importantly, BRCA1 knockdown attenuated SERBP1-driven cisplatin resistance both in vitro and in vivo, establishing BRCA1 as a critical downstream effector of SERBP1. Collectively, these findings identify SERBP1 as a determinant of cisplatin resistance in LUAD and reveal a SERBP1–BRCA1 axis that promotes HR repair and chemoresistance, thereby highlighting SERBP1 as a potential therapeutic target to overcome cisplatin resistance.

## Introduction

Globally, lung cancer is the primary cause of cancer-related death [1]. Among all diagnosed lung cancer cases, 85% are non-small cell lung cancer (NSCLC). Lung adenocarcinoma (LUAD) is the most prevalent subtype of NSCLC [2]. Despite numerous advances in treatments, including targeted therapies and immunotherapies, over the past decade, overall survival and prognosis have not improved for most patients [3]. In the clinic, cisplatin (CDDP), which activates pathways related to apoptosis by triggering DNA damage, is the most commonly used drug for treating NSCLC [4]. However, with the development of chemotherapeutic treatment, the incidence of drug resistance has also increased, limiting the effectiveness of cisplatin in LUAD patients. Currently, the precise mechanisms of cisplatin resistance in NSCLC are unclear. Therefore, there is an urgent need to identify new predictive markers for cisplatin resistance and understand the underlying mechanisms to reveal new treatment targets to improve NSCLC treatment.

SERPINE1 mRNA binding protein 1 (SERBP1) is encoded by the SERBP1 gene, which is also called PAI-1 mRNA binding protein 1 (PAI-RBP1) and is located on chromosome 1p31 [5]. SERBP1 has been reported to regulate the stability of the PAI-1 mRNA by binding to the cyclic nucleotide-responsive sequence (CRS) of the PAI-1 mRNA [6]. The similarity in amino acid sequences indicates that SERBP1 belongs to the HABP4 family and shares a hyaluronan-binding domain and an RNA-binding motif [7]. As a conserved RNA-binding protein, SERBP1 consists of arginine-glycine (RG) and arginine-glycine-glycine (RGG) repeat motifs, which are needed for mRNA targeting. Previous studies have suggested that SERBP1 interacts with nuclear proteins such as CHD3 and Daxx, while SERBP1 is mainly localised in the cytoplasm [8]. SERBP1 is involved in physiological processes in cells, such as the DNA damage response [9]. Moreover, SERBP1 is involved in tumorigenesis and chemoresistance. Previous studies have reported that SERBP1 is overexpressed in various cancers, such as ovarian carcinoma, acute lymphoblastic leukaemia, glioblastoma, breast cancer and lung squamous cell carcinoma [10–14]. Correspondingly, the overexpression of SERBP1 is related to a poor prognosis in tumour patients [15]. Various human cancer cell lines that exhibit cisplatin resistance also display elevated levels of SERBP1 [16]. Nevertheless, the functional target RNAs of SERBP1 are poorly understood. Moreover, there has been little research on the functions and mechanisms of SERBP1 in LUAD chemoresistance, including cisplatin resistance.

Breast cancer susceptibility gene 1 (BRCA1) functions as a tumour suppressor, negatively regulates tumour growth and is strongly associated with familial cancers [17]. The BRCA1 protein participates in a variety of biological processes in cells, such as DNA damage repair, chromatin remodelling, transcription and apoptosis, to maintain chromosomal stability and tumour repression [18, 19]. During the process of DNA damage repair, BRCA1 plays a crucial role in initiating the repair of double-strand breaks (DSBs). Platinum, such as cisplatin, has been shown to be linked to DNA DSBs by inducing DNA cross-linking, which inhibits DNA synthesis and results in cell death [20]. Homologous recombination (HR) repair can be induced by BRCA1, which is an essential system required for DNA DSBs [21]. BRCA1 promotes the recruitment of the recombination-mediated repair protein RAD51 to DNA damage sites [22]. Accordingly, BRCA1 also participates in promoting the chemoresistance of cancer cells [23]. Studies have also revealed a link between elevated BRCA1 expression and the reduced effectiveness of neoadjuvant cisplatin-based chemotherapy in bladder cancer [24]. To date, the exact mechanisms that regulate the effects of BRCA1 on HR repair and chemoresistance are not well understood.

In our study, we reported that SERBP1 is correlated with cisplatin resistance and poor survival in LUAD patients. Mechanistically, SERBP1 increases BRCA1 mRNA stability to increase BRCA1 protein expression and HR repair, which induces cisplatin resistance in LUAD. The interaction between the SERBP1 protein and BRCA1 mRNA suggests that SERBP1 plays a significant role in LUAD chemoresistance.

## Results

### Abnormally high expression of SERBP1 predicts a poor prognosis in LUAD

**Fig. 1:**
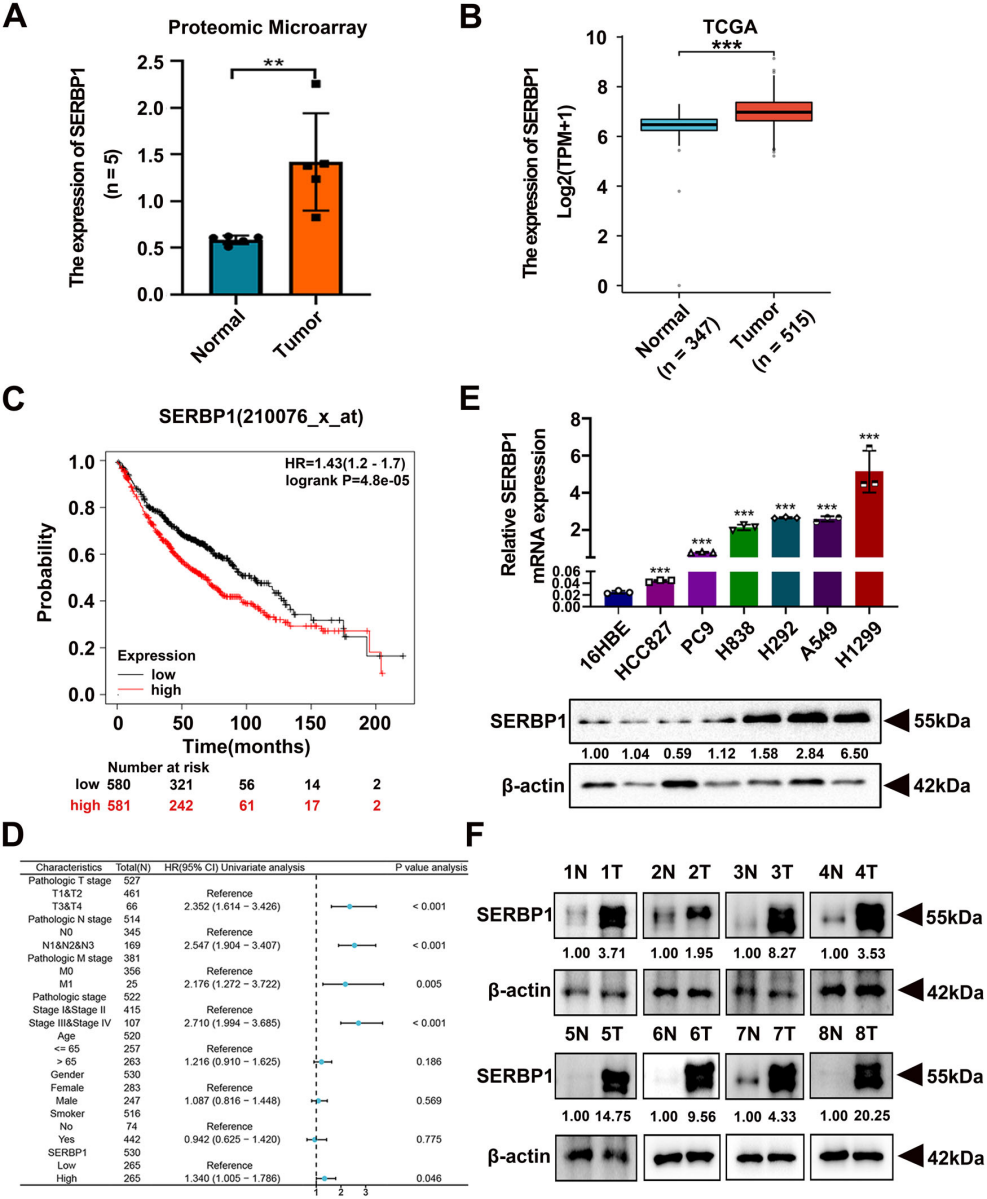
Abnormally high expression of SERBP1 predicts a poor prognosis and is involved in cisplatin resistance in LUAD. **A** The expression of the SERBP1 protein in 5 NSCLC patient tissues compared to that in paired para-cancerous tissues was examined via a quantitative proteomic microarray. **B** SERBP1 expression in the TCGA NSCLC cohort. **C** Effect of the SERBP1 mRNA expression level on overall survival in 1161 NSCLC patients. Kaplan–Meier plots were generated using Kaplan–Meier Plotter. **D** The results from the multivariate analysis of LUAD patients based on the Cox regression model are displayed. Some factors associated with LUAD patient clinical outcomes were introduced to the model. **E** SERBP1 mRNA and protein expression in NSCLC cell lines and normal bronchial epithelial (16HBE) cells. **F** Western blot analysis of protein levels of SERBP1 in 8 paired LUAD and adjacent tissues. Data are shown as the mean ± SD. *P < 0.05; **P < 0.01; ***P < 0.001.

Since the results of our quantitative proteomic microarray revealed that the expression of SERBP1 was higher in 5 paired LUAD patient tissues than in paired paracancerous tissues (Fig. 1A), we further investigated data from The Cancer Genome Atlas (TCGA) public database and found that the expression of SERBP1 protein in NSCLC patients was significantly upregulated (Fig. 1B). Moreover, analysis of a dataset extracted from Kaplan‒Meier plotter revealed that SERBP1 upregulation was associated with shorter overall survival (OS) times in LUAD patients (Fig. 1C). We also evaluated the associations between SERBP1 expression and clinicopathological variables in LUAD patients by using the TCGA online database. As shown in Fig. 1D, increased expression of SERBP1 indicated a higher risk of lymph node metastasis, more severe clinical stage grade and a poorer prognosis. In addition, we detected mRNA and protein expression in 6 NSCLC cell lines along with a normal bronchial epithelial cell line, 16HBE, by qRT‒PCR and western blot analysis. Consistent with the database results, the SERBP1 mRNA and protein expression levels in the NSCLC cell lines were significantly greater than those in the normal cell samples (Fig. 1E). Finally, the results of our western blotting analysis in 8 paired LUAD and adjacent normal tissues also revealed greater expression of SERBP1 in LUAD tissues (Fig. 1F). The findings above strongly suggested that high expression of SERBP1 predicts a poor prognosis and plays a vital oncogenic role in LUAD.

### SERBP1 promotes cisplatin resistance in LUAD

**Fig. 2:**
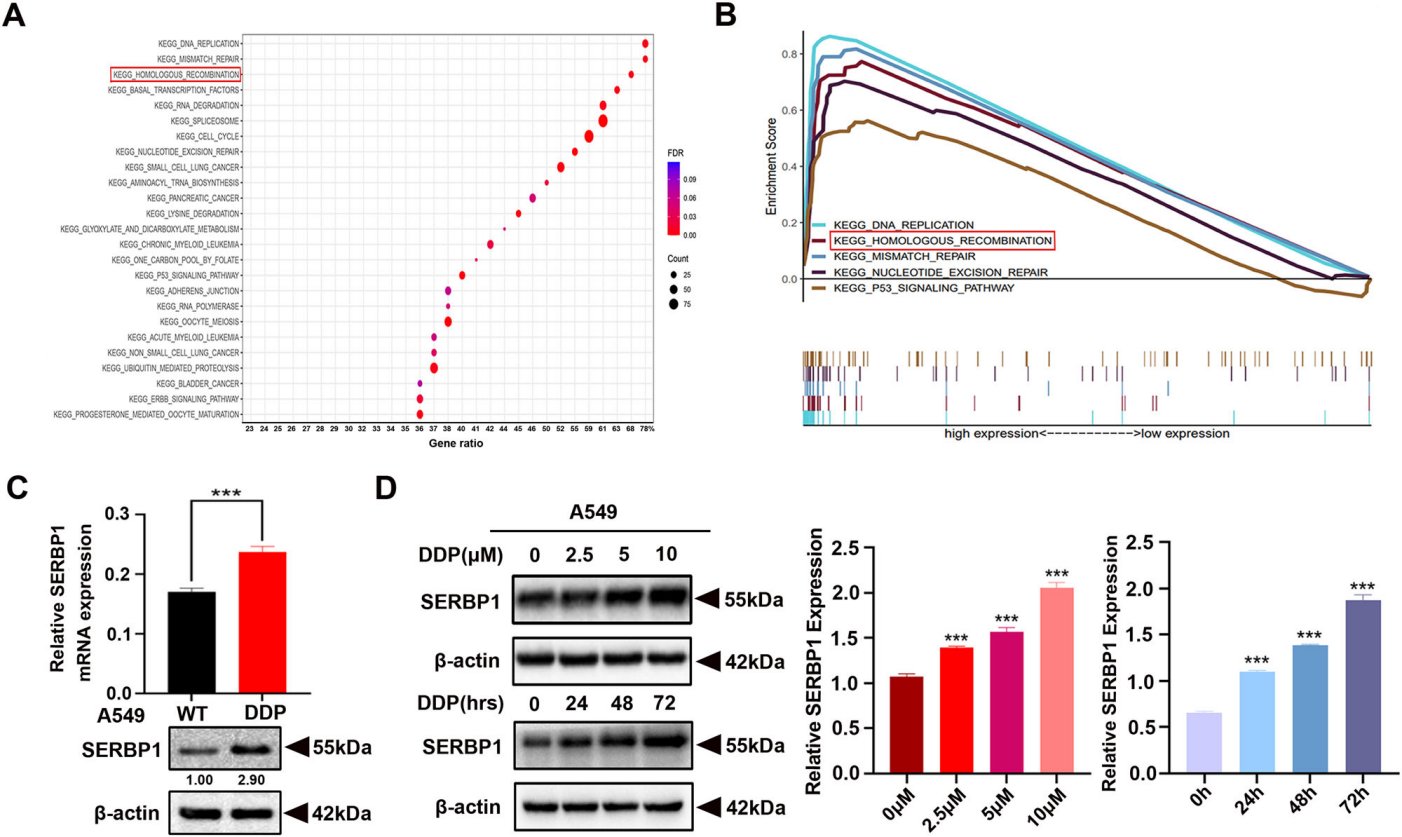
SERBP1 promotes cisplatin resistance in LUAD. **A** KEGG analysis revealed that SERBP1 overexpression was related to enrichment of signalling pathways related to the DNA damage repair. The data were downloaded from the TCGA database. **B** GSEA of the TCGA dataset showed that high expression of SERBP1 was associated with the homologous recombination signalling pathway. **C** The expression level of SERBP1 in A549 and A549/DDP cells was determined by quantitative real-time PCR and western blot analysis. **D** Western blot analysis was used to detect the effect of cisplatin treatment at different concentrations (upper panel) and for different durations (lower panel) on SERBP1 expression in A549 cells. Data are shown as the mean ± SD. ***P < 0.001.

To explore the specific mechanism through which SERBP1 exerts oncogenic functions in LUAD, KEGG pathway analysis was performed. SERBP1 is closely related to DNA damage repair pathways, including DNA replication, mismatch repair and homologous recombination (Fig. 2A). Enrichment score analysis also revealed enrichment of DNA damage repair pathways in cells with high expression levels of SERBP1 (Fig. 2B). SERBP1 reportedly affects homologous recombination in response to DNA double-strand breaks (DSBs). Thus, we hypothesised that SERBP1 induces cisplatin resistance via the homologous recombination signalling pathway. To determine whether there is a relationship between SERBP1 expression and cisplatin resistance, we evaluated SERBP1 expression in cisplatin-sensitive cells (A549, PC9) and cisplatin-resistant cells (A549/DDP, PC9/DDP). DDP cells exhibited elevated SERBP1 expression (Figs. 2C, S5A). Furthermore, LUAD cells (A549 cells) sensitive to cisplatin were exposed to cisplatin at gradually increasing concentrations for 24 h. Moreover, we treated A549 cells with 5 μM cisplatin for different durations. As expected, SERBP1 expression in A549 cells significantly increased after cisplatin treatment in a dose- and time-dependent manner (Fig. 2D), which was consistent with the findings in A549 cells treated with carboplatin (Fig. S1A, B). Therefore, further studies were performed using A549 and A549/DDP cells.

### SERBP1 knockdown sensitises cisplatin resistance in LUAD cells

**Fig. 3:**
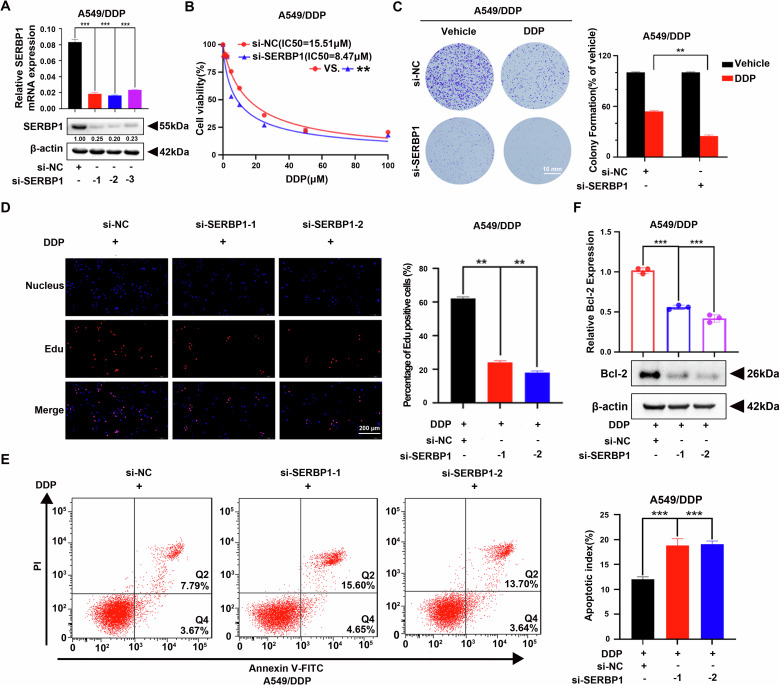
SERBP1 knockdown sensitises cisplatin-resistant LUAD cells to cisplatin. **A** SERBP1 mRNA and protein expression was decreased in SERBP1-knockdown A549/DDP cells. **B** The sensitivity of A549/DDP cells transfected with si-SERBP1 or si-NC to 48 h of cisplatin treatment was determined via CCK-8 assays. **C** Representative images of the colony formation assay results showing the proliferation of SERBP1-knockdown A549/DDP cells treated with vehicle (PBS) or 5 μM cisplatin for 48 h; the data are presented below. Scale bar: 10 mm. **D** Results from the EdU assays of si-NC, si-SERBP1-1 and si-SERBP1-2 cells in the presence of cisplatin (5 µM). Scale bar: 200 μm. **E** Flow cytometry assay of the designated cells that were treated with 5 µM cisplatin for 48 h and stained with Annexin V-FITC and PI. The right bar graphs display the statistical analysis results (right panel). **F** The expression of the apoptosis-related protein Bcl-2 was determined by western blotting. Data are shown as the mean ± SD. **P < 0.01; ***P < 0.001.

SERBP1 was knocked down in DDP cells to construct a loss-of-function model. SERBP1 mRNA and protein expression was significantly reduced after the transfection of DDP cells with three siRNAs against SERBP1 (Fig. 3A, S3A). The CCK-8 assay clearly showed that SERBP1 knockdown increased the cisplatin sensitivity of DDP cells treated with different doses of cisplatin (Fig. 3B, S3B). Moreover, SERBP1 knockdown increased the carboplatin sensitivity of A549/DDP cells (Fig. S1C). Colony formation assays also demonstrated that the number of colonies was significantly decreased in SERBP1-knockdown DDP cells after cisplatin treatment (Fig. 3C, S3C). EdU assays also revealed that SERBP1 knockdown suppressed the proliferation of cisplatin-resistant LUAD cells in vitro (Fig. 3D, S3D), and this effect was more pronounced in cells treated with cisplatin than in those not treated with cisplatin (Fig. S2A). Then, we used flow cytometric analysis and western blotting to evaluate the role of SERBP1 in cisplatin-induced apoptosis. In line with the previous results, the percentage of apoptotic DDP cells increased rapidly after treatment with cisplatin (Fig. 3E, S3E). Western blot analysis further verified decreased expression of Bcl-2, which is an anti-apoptotic protein (Fig. 3F).

### SERBP1 overexpression leads to cisplatin resistance in LUAD cells

**Fig. 4:**
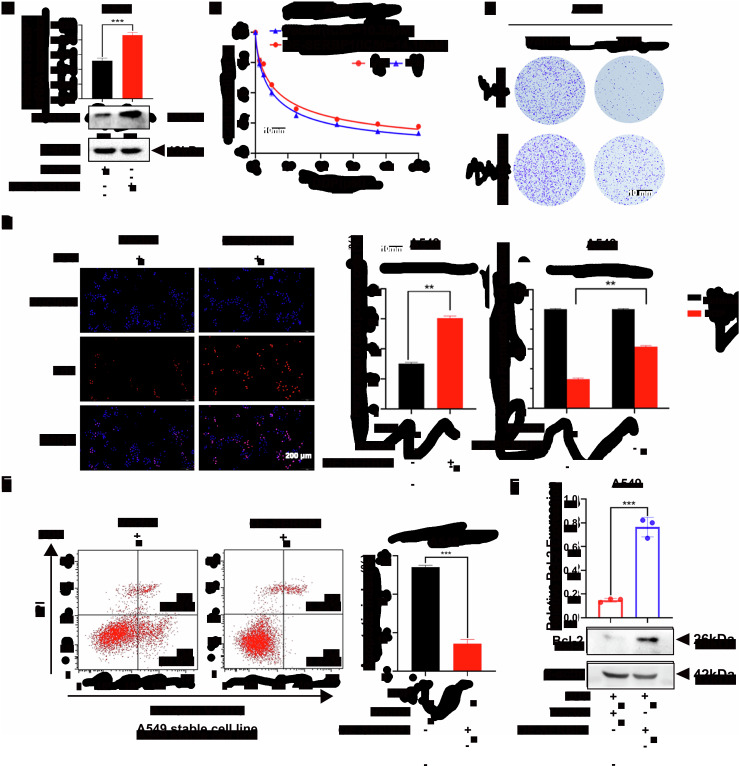
Overexpression of SERBP1 leads to cisplatin resistance in LUAD cells. **A** SERBP1 mRNA and protein expression was increased in SERBP1-overexpressing A549 cells. **B** The sensitivity of SERBP1-overexpressing A549 cells to 48 h of cisplatin treatment was determined by CCK-8 assays. **C** Representative images of the colony formation assay results showing the proliferation of SERBP1-overexpressing A549 cells treated with vehicle (PBS) or 5 μM cisplatin for 48 h. Scale bar: 10 mm. **D** Results from the EdU incorporation assays of vector-transfected and OE-SERBP1 cells in the presence of cisplatin (5 µM). Scale bar: 200 μm. **E** Flow cytometry assay of the designated cells that were treated with 5 µM cisplatin for 48 h and stained with Annexin V-FITC and PI. The right bar graphs display the statistical analysis (right panel). **F** The expression of the apoptosis-related protein Bcl-2 was determined via western blotting. Data are shown as the mean ± SD. **P < 0.01; ***P < 0.001.

We also stably overexpressed SERBP1 in cisplatin-sensitive cell line to establish a gain-of-function model. We verified the increased mRNA and protein expression levels by qRT‒PCR and western blotting (Fig. 4A, S4A). CCK-8 assays revealed that SERBP1 overexpression promoted chemoresistance in A549 cells (Fig. 4B, S4B). As expected, SERBP1 overexpression significantly increased the carboplatin sensitivity of the A549 cell line (Fig. S1D). Colony formation (Figs. 4C, S4C) and EdU (Figs. 4D, S4D) assays revealed that SERBP1 overexpression promoted the proliferation of cisplatin-sensitive LUAD cells in vitro, and this effect was more obvious in the presence of cisplatin than in the absence of cisplatin (Fig. S2B). Furthermore, flow cytometric analysis indicated that high expression of SERBP1 decreased apoptosis (Figs. 4E, S4E). Moreover, the expression of the apoptotic marker Bcl-2 increased, as detected by western blotting (Fig. 4F). These results strongly suggested that SERBP1 plays a vital role in promoting cisplatin resistance in LUAD cells.

### SERBP1 promotes cisplatin resistance via the homologous recombination signalling pathway in LUAD

**Fig. 5:**
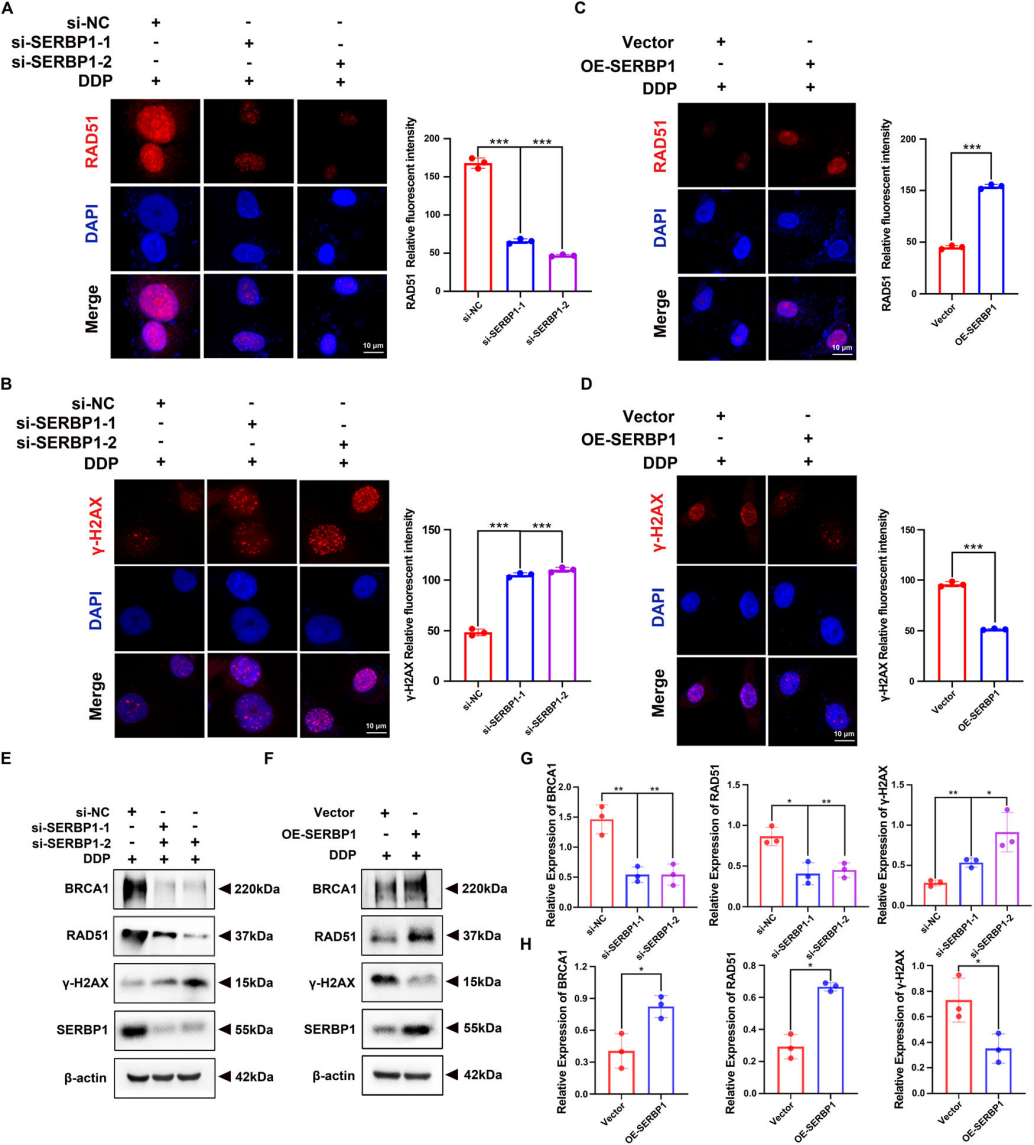
SERBP1 promotes cisplatin resistance via the homologous recombination signalling pathway in LUAD. **A**, **C** Representative immunofluorescence images showing RAD51 expression in SERBP1-knockdown A549/DDP cells and SERBP1-overexpressing A549 cells. **B**, **D** Representative immunofluorescence images showing γ-H_2_AX expression in SERBP1-knockdown A549/DDP cells and SERBP1-overexpressing A549 cells. Scale bar: 10 μm. **E**, **F**, **G**, **H** Western blot analysis was used to verify that SERBP1 regulated HR-related proteins, including BRCA1, RAD51, and γ-H_2_AX, in LUAD. Data are shown as the mean ± SD. *P < 0.05; **P < 0.01; ***P < 0.001.

To verify this speculation, immunofluorescence assays were applied to measure the formation of RAD51 and γ-H_2_AX foci in A549/DDP cells treated with cisplatin for 24 h. As expected, the number of RAD51 foci decreased after SERBP1 was knocked down in cisplatin-resistant cells (Fig. 5A). Correspondingly, the number of γ-H_2_AX foci, which indicate DNA damage, increased (Fig. 5B). Conversely, SERBP1 overexpression significantly increased RAD51 foci formation (Fig. 5C) and reduced the number of γ-H_2_AX foci (Fig. 5D). Western blot analysis further confirmed that knockdown of SERBP1 inhibited the expression of BRCA1 and RAD51, which play important roles in HR repair. Consistent with these findings, γ-H_2_AX protein expression increased (Figs. 5E, G, S5B). SERBP1 overexpression had the opposite effect (Figs. 5F, H, S5C). These data indicated that SERBP1 promotes cisplatin resistance via the homologous recombination signalling pathway in LUAD.

### SERBP1 binds to BRCA1 mRNA and increases its stability

**Fig. 6:**
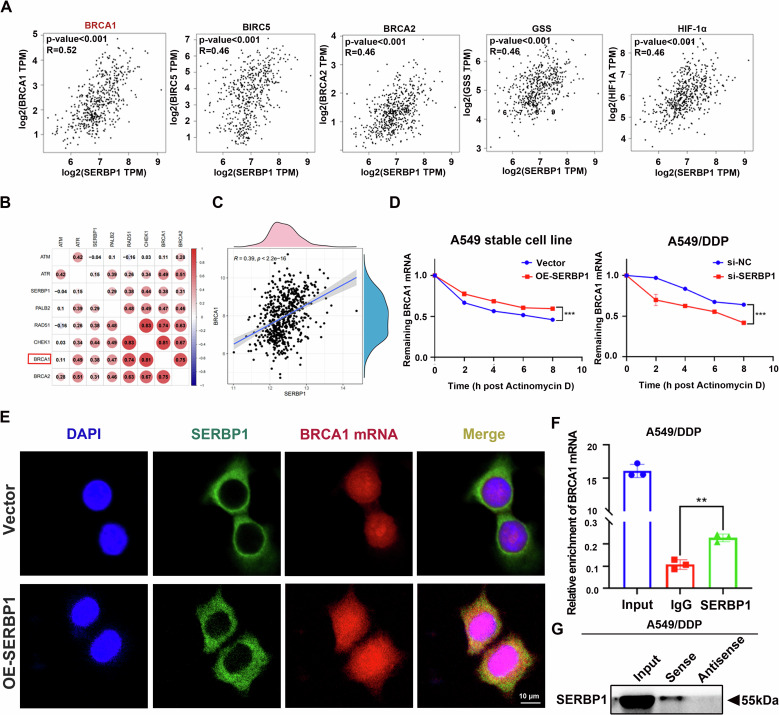
SERBP1 binds to BRCA1 mRNA and regulates its stability. **A** Correlations between SERBP1 and critical molecules related to cisplatin resistance were evaluated in the GEPIA database. **B** Correlation analysis between SERBP1 and HR-related molecules was conducted with TCGA data. **C** TCGA database analysis revealed a relationship between the expression of SERBP1 and BRCA1 in LUAD samples. **D** The relative mRNA expression ratio of BRCA1 was analysed by qRT‒PCR in actinomycin D-treated A549 and A549/DDP cells at various time points. **E** FISH-IF assays showing the localisation and expression of SERBP1 and BRCA1 mRNA in A549 stable cell lines. Scale bar: 10 μm. **F** RIP assays were performed using an anti-SERBP1 antibody or IgG to detect binding to BRCA1 mRNA in A549/DDP cells. IgG was used as the negative control. **G** RNA pull-down assays were applied to detect the expression of SERBP1 using beads containing BRCA1 mRNA. Data are shown as the mean ± SD. **P < 0.01; ***P < 0.001.

Next, we sought to understand the underlying mechanism responsible for SERBP1-mediated homologous recombination repair and LUAD cisplatin resistance. According to previous publications, the BRCA1, BIRC5, BRCA2, GSS and HIF1A genes are the most closely associated with cisplatin resistance [25–29]. In this respect, bioinformatics analysis was employed to evaluate the association of these genes with SERBP1. Notably, BRCA1 displayed the closest association with SERBP1 (Fig. 6A, B, C). Moreover, BRCA1 is involved in activating DNA HR repair [21]. SERBP1 functions as an RNA-binding protein. Overall, we hypothesised that SERBP1 promotes BRCA1 mRNA stability by binding to it, subsequently inducing HR repair-mediated cisplatin resistance in LUAD. As shown in the graph, the stability of BRCA1 mRNA increased after SERBP1 overexpression in cisplatin-sensitive cells. Similarly, in SERBP1-knockdown DDP cells, we observed decreased BRCA1 mRNA expression (Figs. 6D, S5D). These results indicated that SERBP1 can increase the stability of BRCA1 mRNA, leading to promote BRCA1 protein expression in DNA damage repair signalling pathways. To further elucidate how SERBP1 directly or indirectly interacts with BRCA1 mRNA, we first carried out molecular docking between SERBP1 and BRCA1 mRNA for tertiary structure prediction via NPDock (Fig. S2C). Then, we performed FISH-IF assays via confocal laser scanning microscopy. SERBP1 and BRCA1 mRNA were colocalized in the cytoplasm of A549 LUAD cells (Fig. 6E). In addition, the overexpression of SERBP1 implied a tighter integration of SERBP1 and BRCA1 mRNA. We also carried out RIP assays with antibodies against SERBP1 (Fig. 6F). In addition, RNA pull-down assays using beads containing BRCA1 mRNA were used to validate this assumption (Fig. 6G). The last three experiments verified the direct interaction of SERBP1 and BRCA1 mRNA. In conclusion, SERBP1 binds to BRCA1 mRNA directly and increases its stability.

### BRCA1 knockdown blocks SERBP1-induced cisplatin chemoresistance

**Fig. 7:**
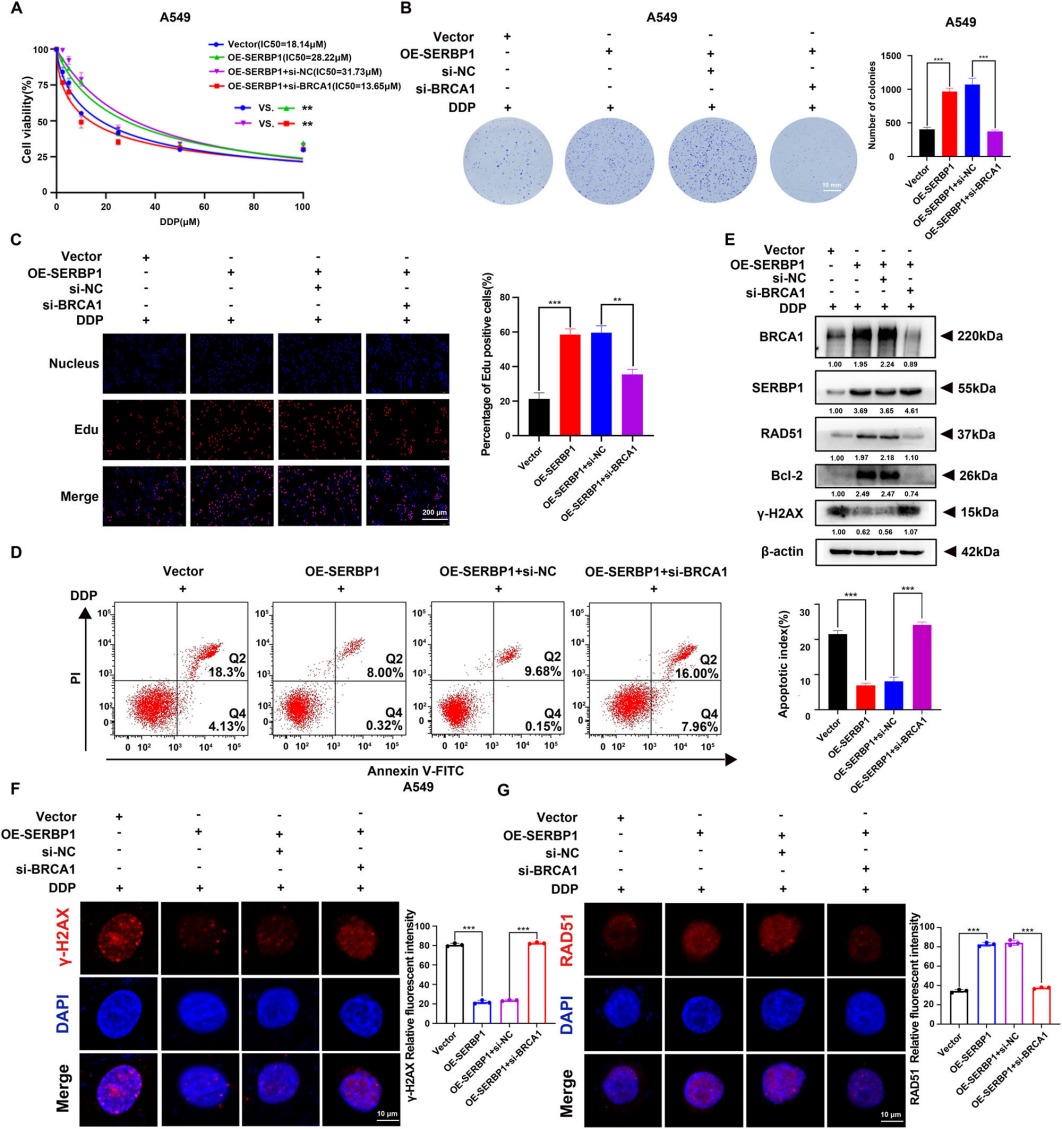
BRCA1 knockdown blocks SERBP1-induced cisplatin chemoresistance. **A** CCK-8 assays were used to assess the viability of SERBP1-overexpressing A549 cells transfected with siRNAs targeting BRCA1 or with si-NC and treated with different concentrations of cisplatin. **B** Colony formation assays were performed to assess the proliferation ability of the A549 stable cell lines after treatment with cisplatin (5 µM) for 48 h. Scale bar: 10 mm. **C** EdU incorporation assays of the A549 stable cell lines in the presence of cisplatin (5 µM). Scale bar: 200 μm. **D** Flow cytometry assay of the designated cells that were treated with 5 µM cisplatin for 48 h and stained with Annexin V-FITC and PI. The right bar graphs display the statistical analysis results (right panel). **E** Western blotting results showing the protein expression of the HR repair marker RAD51 and the DNA damage marker γ-H_2_AX. **F** Representative immunofluorescence images showing γ-H_2_AX expression in the designated cells. **G** Representative immunofluorescence images showing RAD51 expression in the designated cells. Scale bar: 10 μm. Data are shown as the mean ± SD. **P < 0.01; ***P < 0.001.

Previous studies have shown that BRCA1 is involved in the chemoresistance of cancer cells [23]. Therefore, we extended our study to investigate the function of BRCA1 knockdown in SERBP1-induced cisplatin chemoresistance, which revealed the vital role of BRCA1 in this mechanism. We transfected si-BRCA1 into SERBP1-overexpressing A549 cells. Then, we confirmed the knockdown efficacy by western blotting (Fig. 7D). CCK-8 (Fig. 7A), colony formation (Fig. 7B) and EdU (Fig. 7C) assays were used to assess cell proliferation after cisplatin treatment. In line with our expectations, knockdown of BRCA1 attenuated the hyperproliferation of cells induced by SERBP1 overexpression. Moreover, flow cytometry revealed that BRCA1 depletion inhibited SERBP1-induced apoptosis (Fig. 7D). These effects were more obvious in the presence of cisplatin than in the absence of cisplatin (Fig. S2D-E). Moreover, IF assays and western blotting revealed that the SERBP1-mediated increase in the expression of the HR repair marker RAD51 and decrease in the expression of the DNA damage marker γ-H_2_AX were attenuated by BRCA1 knockdown (Fig. 7E, F, G). These results all suggest that BRCA1 is required for SERBP1-mediated cisplatin chemoresistance and that its depletion can attenuate cisplatin chemoresistance induced by SERBP1 overexpression.

### SERBP1 promotes cisplatin resistance by promoting BRCA1 function in HR repair in vivo

**Fig. 8:**
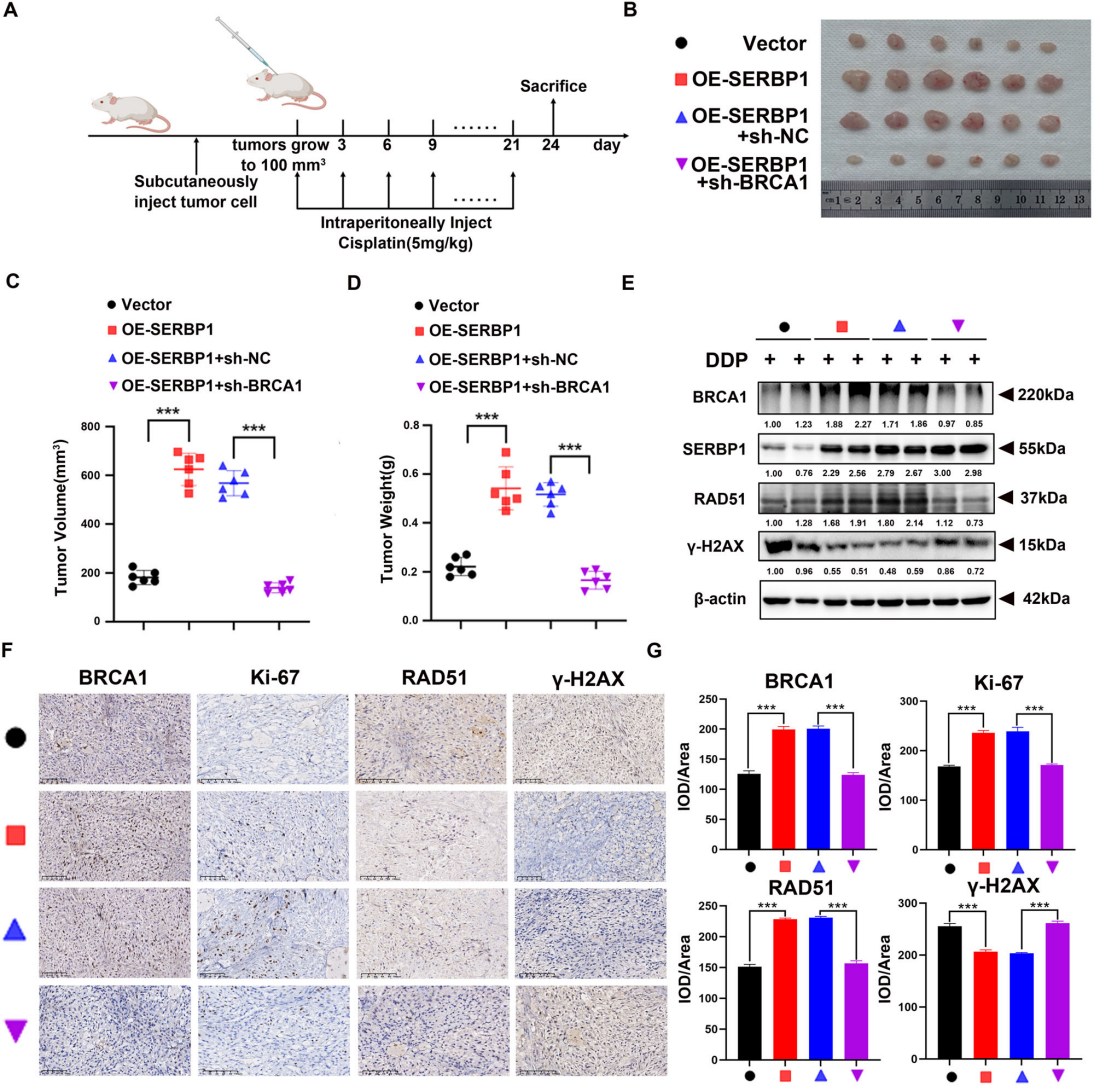
SERBP1 promotes cisplatin resistance by promoting BRCA1 function in HR repair in vivo. **A** flowchart of the in vivo LUAD cell model. A549 stable cells (3 × 10^6^ cells/mouse) were injected subcutaneously into BALB/c nude mice (*n* = 6 mice per group). Cisplatin was administered intraperitoneally (5 mg/kg) to the mice once the tumours grew to 100 mm^3^ every 3 days for eight times. **B** Xenograft tumours were dissected and photographed. **C** The tumour volume was statistically analysed. **D** Tumour weights were measured after the mice were sacrificed. **E** Western blotting was used to analyse the protein levels of RAD51 and γ-H_2_AX in the tumour tissues from the different groups. **F**, **G** IHC analysis of the indicated proteins in xenograft tumours. Scale bar: 100 μm. Data are shown as the mean ± SD. ***P < 0.001.

To further explore the evidence confirming that SERBP1 induces cisplatin resistance by promoting BRCA1 function in HR repair in vivo, we injected stable A549 cells into subcutaneous sites in the flanks of immunocompromised BALB/c nude mice to establish an in vivo model. Nude mice were randomly divided into four groups, namely, the Vector, OE-SERBP1, OE-SERBP1+sh-NC, and OE-SERBP1+sh-BRCA1 groups, which were separately transplanted with the indicated cells as described above. Cisplatin was administered to mice intraperitoneally (5 mg/kg) every 3 days once subcutaneous tumours grew to 100 mm^3^. These cells were treated with cisplatin eight times (Fig. 8A). Tumours were measured and weighed after the mice were sacrificed. Compared with mice injected with Ctrl cells, mice injected with A549 cells overexpressing SERBP1 exhibited increased tumour growth. As expected, BRCA1 knockdown decreased SERBP1-mediated tumour growth, as reflected by the changes in tumour size (Fig. 8B), tumour volume (Fig. 8C), and tumour weight (Fig. 8D). Western blotting and IHC assays also suggested that the expression levels of RAD51 and Bcl-2 in tumours from the group overexpressing SERBP1 were significantly greater than those in tumours derived from Ctrl cells. Similarly, the expression levels of γ-H_2_AX, which indicate the extent of DNA damage, were greatly reduced. BRCA1 depletion prevented the effect of SERBP1 overexpression in response to cisplatin, as illustrated by RAD51 and γ-H_2_AX protein expression (Fig. 8E–G). Overall, in vivo experiments comprehensively verified that SERBP1 induced cisplatin chemoresistance via HR repair in a BRCA1-dependent manner in LUAD.

## Discussion

Most NSCLC patients receive an extensive treatment regimen consisting of chemotherapy, radiotherapy, immunotherapy, targeted therapy or a combination of these methods, which ultimately depends on patient status and disease stage [30]. Despite progress in therapeutic strategies, overall survival and prognosis have not improved significantly. For NSCLC patients, conventional chemotherapy drugs, especially cisplatin, remain the standard treatment. Notably, the emergence of chemoresistance poses a main obstacle to the treatment of NSCLC patients. Thus, it is crucial to reveal the underlying mechanisms of chemoresistance in NSCLC. To date, multiple mechanisms have been reported to confer cisplatin resistance [31]. Cisplatin resistance can be attributed to three main molecular mechanisms. The first is increased DNA repair; for example, increased expression of the nucleotide excision repair-related protein ERCC1 is associated with cisplatin resistance [32]. The second mechanism is altered cellular accumulation. Copper uptake protein 1 (CTR1) induces acquired cisplatin resistance in this way [33]. The third is the downregulated expression of antiapoptotic proteins, such as Bcl-2 [34]. The last mechanism is the alteration of key molecular signalling pathways, such as the IGF and EGFR pathways [35, 36]. Here, SERBP1 was recognised as an innovative biomarker for predicting cisplatin resistance in LUAD.

In the present study, our team first reported that the expression of SERBP1 increased in cisplatin-resistant LUAD cell lines. Then, we found that SERBP1 overexpression led to cisplatin resistance in LUAD cells. Furthermore, SERBP1 binds to BRCA1 mRNA and regulates its stability, which activates the homologous recombination signalling pathway to facilitate DNA repair. In the process of HR, BRCA1 also recruits RAD51 to form multimeric nucleoprotein complexes, eventually inducing cisplatin resistance in LUAD cells [37]. We combined bioinformatics analysis, cell models and animal experiments to confirm our findings.

Previous studies have demonstrated that as an RNA-binding protein, SERBP1 regulates gene expression by impacting vital pathways involved in tumour initiation and growth. SERBP1 was reported to be a target of the tumour suppressor mi-218 in hepatocellular carcinoma (HCC) that is related to tumour cell migration and invasion [36]. Moreover, SERBP1 was found to be a novel oncogenic factor and a potential therapeutic target for glioblastoma [12]. SERBP1 also participates in regulating translation through binding to ribosomal proteins [38]. Recent research revealed that SERBP1 exhibited a protective effect on germ cell viability against heat shock stress by regulating arsenite- and heat shock-induced stress granule elimination [39]. In this study, we found that SERBP1 has profound value as a practical target for predicting cisplatin sensitivity. Our findings provide new ideas for overcoming cisplatin resistance.

Remarkably, SERBP1 expression significantly increased after cisplatin treatment in a dose- and time-dependent manner. On the other hand, studies have shown that RNA methyltransferase 3 (METTL3) and N6-methyladenosine modification of DNA damage-associated RNAs may facilitate homologous recombination-mediated DNA damage repair after cisplatin-induced double-strand breaks (DSBs) [40]. Moreover, YTHN6-methyladenosine RNA binding protein 1 (YTHDF1), which is an N6-methyladenosine modification (m6A) reader, reduces DSB DNA damage and induces chemoresistance [41]. It has been shown that protein arginine N-methyltransferase 1 (PRMT1) promotes protein arginine methylation of SERBP1, affecting protein interactions and intracellular localisation [42]. Hence, we conducted bioinformatics analysis to confirm whether the upregulated expression of SERBP1 induced by cisplatin was closely related to YTHDF1 or other m6A-related enzymes. As expected, the results were consistent with our hypothesis. However, further research is needed to fully support our hypothesis, contributing to a deeper understanding of the role of SERBP1 in the cisplatin resistance of LUAD.

Recently, many new therapeutic strategies have been explored to address cisplatin resistance in various cancers. For instance, the gene heterogeneous nuclear ribonucleoprotein U (HNRNPU) was discovered to be the top candidate gene responsible for cisplatin resistance in bladder cancer. Inhibition of HNRNPU might be a possible therapy for bladder cancer that is resistant to cisplatin [43]. In another study, ubiquitin-specific peptidase 1 (USP1) was shown to regulate the development of microtubule-associated serine/threonine kinase 1 (MAST1)-mediated cisplatin resistance in cancers. Thus, a novel therapeutic strategy targeting both these molecules could increase cisplatin efficacy in tumours [44]. Beatrice et al. reported that inhibition of mitochondrial HSP60 (HSPD1) could increase chemotherapy drug sensitivity in cancers by impairing mitochondrial metabolism [45]. Finally, Chintan et al. reported that targeting the tumour microenvironment attenuated chemoresistance for the effective management of cancer [46]. However, no relevant inhibitors have been approved for clinical use on the basis of our present knowledge of chemoresistance mechanisms. In our study, SERBP1 affects cisplatin resistance. From a clinical perspective, it is necessary to develop a specific inhibitor against SERBP1. The combination of a SERBP1-specific inhibitor and cisplatin is expected to induce synergistic effects to greatly increase cisplatin efficacy, satisfying the unmet clinical need related to cisplatin resistance.

## Conclusion

**Fig. 9:**
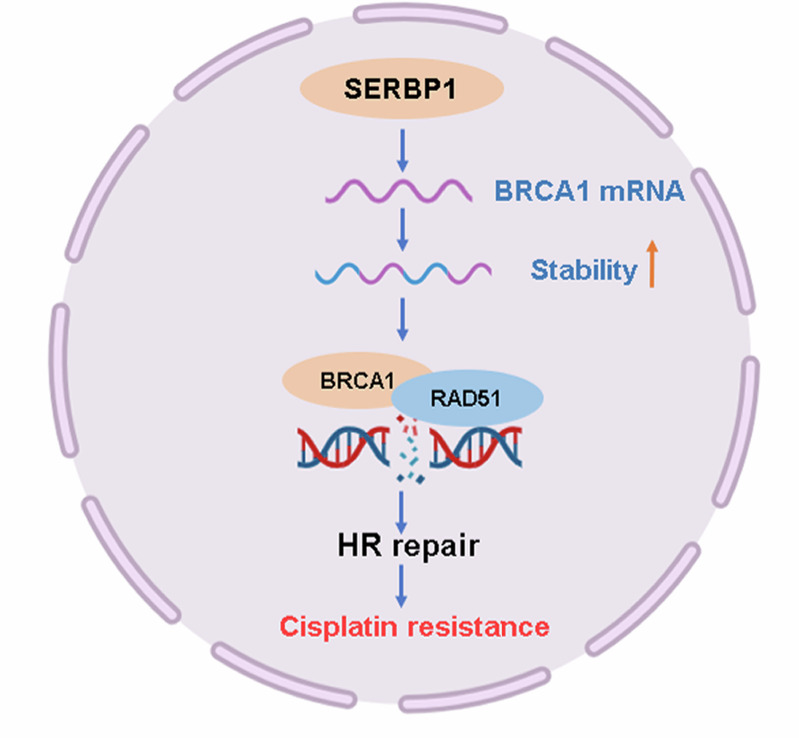
Graphical diagram of SERBP1 promotes cisplatin chemoresistance in lung adenocarcinoma by increasing BRCA1 mRNA stability in HR repair. SERBP1 is upregulated in cisplatin-resistant LUAD cells and predicts a poor prognosis in LUAD patients. SERBP1 promotes cisplatin resistance via the homologous recombination signalling pathway in LUAD. BRCA1 knockdown can rescue SERBP1-induced cisplatin chemoresistance. Mechanistically, SERBP1 stabilises BRCA1 mRNA, thus recruiting RAD51 and promoting HR repair in LUAD cells.

A schematic diagram summarising the findings of our study is shown in Fig. 9. This new evidence highlights promising therapeutic targets for cisplatin-resistant LUAD that could be applied in the clinic.

## Materials and methods

### Cell culture

The human NSCLC cell lines A549 and A549/DDP (a lung adenocarcinoma cell line resistant to cisplatin) were purchased from the Pricella Life & Technology Co., Ltd. (Wuhan, China). Other NSCLC cell lines (H1299, H292, H838, PC9, HCC827) and a normal bronchial epithelial cell line, 16HBE were purchased from the Cell Bank of the Chinese Academy of Sciences (Shanghai, China). A549 and A549/DDP were grown in F12K Medium (Gibco, Carlsbad, CA, USA). Other tumour cells were cultivated in RPMI 1640 medium. All cell lines were supplemented with 10% foetal bovine serum (FBS) (Gibco, Carlsbad, CA, USA) at 37 °C in a humidified air atmosphere containing 5% CO_2_. All cell lines we used were authenticated by STR profiling.

### RNA interference

Predesigned small interfering RNA (siRNA) sequences targeting different coding regions of SERBP1 or BRCA1 were directly synthesised by GenePharma (Suzhou, China). The target sequences of the siRNAs are listed in Supplementary Table 2. Scrambled siRNA was used as a negative control. Cells were transiently transfected with 100 pmol of siRNA sequences using Lipofectamine 2000 (Invitrogen, Carlsbad, CA, USA). After 72 h of transfection, the cells were harvested for further experiments.

### Establishment of stable SERBP1-overexpressing cell lines

The SERBP1-overexpressing plasmid was purchased from Banma Biotechnology Co., Ltd. (Changsha, China). A negative control was provided via the empty vector. Human embryonic kidney 293T cells were cultured in Dulbecco’s modified Eagle’s medium (DMEM) supplemented with 10% FBS at 37 °C in a humidified 5% CO_2_ incubator for 48 h. After incubation, the packaged lentiviruses were collected and used to infect A549 cells. Stable cells were selected with 2 μg/ml puromycin (Sigma-Aldrich, St Louis, MO, USA) after 48 h.

### Establishment of stable BRCA1-silenced cell lines

To construct stable cell lines with silenced BRCA1 expression, one DNA fragment (BRCA1 shRNA-1, 5′-CCGGGAGTATGCAAACAGCTATAATCTCGAGATTATAG CTGTTTGCATACTCTTTTTG-3′) was subcloned and inserted into the lentiviral vector pLKO.1-U6-Puro (GenePharma, Shanghai, China) containing the endonucleases SwaI and NotI. A scrambled sequence of the BRCA1 shRNA (5′-CCGGCAACAAGATGAAGAGCACCAACTCGAGTTGGTGCTCTTCATCTT GTTGTTTTTG-3′), which served as the negative control, was used. Then, the BRCA1-silenced construct or negative control was cotransfected with packaging plasmids into human embryonic kidney 293T cells using Lipofectamine 2000 (Invitrogen, Carlsbad, CA, USA). After 48 h, the cells were infected with the packaged lentiviruses and cultured for 2 days before being selected with 2 μg/ml puromycin (Sigma-Aldrich, St Louis, MO, USA).

### RNA extraction, cDNA synthesis, and quantitative real-time PCR (qRT‒PCR)

According to the manufacturer’s protocol, total RNA was extracted from cells by the addition of 1 ml of RNAiso Plus (Takara, Japan). The RNA concentration was measured with a NanoDrop 2000 (Thermo Fisher, Waltham, USA). We carried out cDNA synthesis using M-MLV reverse transcriptase (Takara, Japan). The primers used for reverse transcription and amplification of SERBP1 were designed and synthesised by Genewiz (Suzhou, China). The primers for SERBP1 and β-actin used for qRT‒PCR analysis were as follows: SERBP1, forward: 5′-TAGACCGATTATTGACCGACCT-3′ and reverse: 5′-GTTTGCCACGAGAATC AAATCC-3′; β-actin, forward: 5′-CACAGAGCCTCGCCTTTGCC-3′ and reverse: 5′-ACCCATG CCCACCATCACG-3′. qRT‒PCR was performed using SYBR Premix ExTaq™ (Takara, Japan) according to the manufacturer’s instructions with an ABI Step One Plus Real-Time PCR system (Applied Biosystems). The PCR programme was as follows: 95 °C for 10 min, followed by 40 cycles at 95 °C for 15 s and 60 °C for 1 min. The expression value of SERBP1 mRNA was normalised to the value of the internal control β-actin. Relative expression was calculated using the ^ΔΔ^Ct method.

### Western blot analysis and antibodies

Western blot analysis was conducted as previously described [47]. The following antibodies were used in this study: anti-SERBP1 (10729-1-AP), anti-BRCA1 (22362-1-AP) (all from Proteintech, Wuhan, China), anti-Bcl-2 (#4223), anti-γ-H_2_AX (#9718S) (all from Cell Signaling Technology, Danvers, MA, USA), and anti-RAD51 (ab133534) (Abcam, Cambridge, UK). Anti-β-actin (CW0096M), anti-rabbit (CW0103), and anti-mouse (CW0102) secondary antibodies were obtained from Cowin, Beijing, China.

### Cell viability assay

Cells were plated in each well of a 96-well plate at a density of 3000 cells per well, grown overnight and then treated with various drug concentrations for 48 h. Cell viability was determined using Cell Counting Kit-8 (Boster, Wuhan, China) according to the manufacturer’s instructions. The fluorescence at 630 nm and 450 nm was measured using a microplate reader after 1–2 h (Thermo Fisher, Waltham, USA). We also assessed cell proliferation using a colony formation assay. In brief, tumour cells were treated with or without cisplatin (5 μM) for 48 h, and then, 3000 cells were reseeded in a 60-mm plate. After incubation for 7–14 days, depending on the cell growth rate, colonies with at least 50 cells were stained with 0.1% crystal violet and counted. Each experiment was performed in triplicate. The EdU assay kit was purchased from RiboBio Company (C10310-1, Guangzhou, China). We performed the experiments strictly following the kit instructions. An Olympus IX-73 inverted microscope (Tokyo, Japan) was used to obtain images.

### Flow cytometry analysis

According to the protocol of the Cell Cycle Analysis Kit (Beyotime, Shanghai, China), tumour cells were seeded in 6-well plates per well and treated with cisplatin (5 μM) for 48 h. The cells were then harvested, washed with cold PBS, fixed with 70% ethanol at 4 °C for 24 h, washed with cold PBS again and stained with a propidium iodide (PI)/RNase mixture. Next, the cells were incubated in the dark at 37 °C for 30 min and analysed using a fluorescence-activated cell sorting (FACS) Calibur system (BD Canto II, BD Biosciences, New Jersey, USA). For the apoptosis assay, after 48 h, the cells were harvested, washed with cold PBS, and resuspended in binding buffer containing Annexin V/FITC and PI (Beyotime, Shanghai, China). The stained cells were then detected using a FACS Calibur system (BD Canto II, BD Biosciences, New Jersey, USA).

### Immunofluorescence staining

After treatment with cisplatin for 48 h, the cells were seeded in 12-well plates precoated with glass slides for 24 h. At a density of 40–50%, the cells were washed with PBS and then fixed with 4% paraformaldehyde for 30 min, followed by permeabilization with 0.5% Triton X-100 solution for 20 min. After blocking with 5% bovine serum albumin (BSA) for 1 h at room temperature, the cells were incubated overnight with anti-primary antibodies (anti-SERBP1, anti-RAD51, and anti-γH_2_AX) at 4 °C. Then, the cells were incubated with the corresponding secondary antibodies (Alexa Fluor 647-conjugated anti-rabbit IgG, anti-mouse IgG, and FITC Fluor 547-conjugated anti-rabbit IgG) (Beyotime, Shanghai, China) for 1 h in the dark. Finally, the samples were stained with DAPI for 10 min. Images were taken with a Leica SP8 confocal microscope (Leica, Wetzlar, Germany) with optimal settings for the fluorescent markers used.

### Immunohistochemistry assay

Immunohistochemistry (IHC) analyses of tissues were conducted as described in our previous study [48]. In brief, the sections were incubated with BRCA1-, RAD51- and γ-H_2_AX-specific monoclonal primary antibodies overnight at 4 °C, followed by incubation with biotinylated secondary antibodies. The reactions were developed using a DAB Kit (BD Bioscience, New Jersey, USA), and the sections were counterstained with haematoxylin.

### RNA‑binding protein immunoprecipitation (RIP)

RNA‑binding protein immunoprecipitation (RIP) was conducted using a RIP kit (BersinBio, Guangzhou, China) according to the manufacturer’s protocol. The cells were lysed with RIP lysis buffer. Then, we incubated the lysate products with magnetic beads preconjugated to an anti-IgG or anti-SERBP1 antibody at 4 °C overnight. Finally, RNA was extracted and purified to determine the expression level of BRCA1 mRNA by qRT‒PCR.

### RNA stability assays

Tumour cells were treated with 5 μg/ml actinomycin D (Cat# S8964) (Selleck, Houston, TX, USA) for the indicated times. Total RNA was subsequently extracted and processed for qRT‒PCR analysis.

### Bioinformatics analysis

NSCLC patient data were downloaded from the TCGA database (www.cancer.gov). Then, we conducted differential expression analysis according to the data. We performed patient survival analysis with Kaplan‒Meier Plotter (www.kmplot.com). Gene Set Enrichment Analysis (GSEA) 4.1.0 software was used to perform KEGG pathway analysis. Correlation analysis was performed using Gene Expression Profiling Interactive Analysis (GEPIA, www.gepia.cancer-pku.cn). We used R software (4.0.3) to analyse the data.

### Tumour xenograft animal model

Female BALB/c athymic nude mice (4–6 weeks old and weighing 16–20 g) were purchased from the Experimental Animal Center of Soochow University and bred under pathogen-free conditions. All the animal experiments were carried out in accordance with the Guide for the Care and Use of Experimental Animals Center of Soochow University. To establish the lung carcinoma xenograft model, A549 stable cell lines were suspended in 100 ml of RPMI 1640 medium and inoculated subcutaneously into the flanks of nude mice, which were randomly divided into four groups (6 mice in each group). The tumour volume (V) was determined by measuring the tumour length (*L*) and width (*W*) with a Vernier calliper and applying the formula *V* = (*L* × *W*^2^) × 0.5. When the tumour volume reached 100–150 mm^3^, cisplatin was administered via intraperitoneal injection at 5 mg/kg every 3 days until the mice were sacrificed.

### RNA pull-down assay

The BRCA1 3’UTR and an antisense sequence of the wild-type BRCA1 3’UTR were synthesised and labelled using biotin (RiboBio Biotech, Guangzhou, China). An in vitro RNA pull-down assay was performed using the Pierce^TM^ Magnetic RNA‒Protein Pull-down Kit (Thermo Fisher, Waltham, USA) according to the manufacturer’s protocol. In brief, cell lysates were incubated with beads containing biotin-labelled RNAs for 1 h at 4 °C. The bound proteins were eluted and boiled in 1 × SDS lysis buffer and subjected to western blot analysis to detect the presence of SERBP1. Each experiment was performed in triplicate.

### RNA fluorescence in situ hybridisation (RNA-FISH)

A fluorescence in situ hybridisation kit (RiboBio, Guangzhou, China) was used in accordance with the manufacturer’s directions for RNA-FISH analysis. First, the cells were seeded on sterilised glass slides and fixed with 4% paraformaldehyde for 10 min at room temperature. Then, the samples were permeabilized with 0.5% Triton X-100 in PBS for 5 min at 4 °C followed by prehybridization. Then, 200 μl of prehybridization buffer was added to the cells at 37 °C for 30 min. The fixed cells were then hybridised to a 5 mM probe at 37 °C in the dark overnight. The slides were washed three times with Wash Buffer I (4×SSC with 0.1% Tween-20), once with Wash Buffer II (2×SSC), and once with Wash Buffer III (1×SSC) at 42 °C in the dark for 5 min and once with 1×PBS at room temperature. Finally, the cells were stained with 1× DAPI in the dark for 10 min. BRCA1 mRNA-cy3 FISH probes were designed and synthesised by RiboBio Co., Ltd. All images were visualised and obtained via a Leica SP8 confocal microscope (Leica, Wetzlar, Germany) with optimal settings for the fluorescent markers used.

### Statistical analysis

The quantitative variables are presented as the means and SDs. All the statistical analyses were performed using GraphPad Prism 8.0 (GraphPad Software, San Diego, CA, USA). Kaplan-Meier survival curves were created, and Cox regression analysis assessed differences in survival probabilities between groups. Differences between two groups were analysed by an unpaired Student’s *t* test. (two-tailed; *p* < 0.05 was considered to indicate statistical significance). One-way/two-way ANOVA was used to analyse multiple-group comparisons. Results were considered to be statistically significant at *P* < 0.05, with *P < 0.05, **P < 0.01, ***P < 0.001.

### Ethics approval

The author confirms that all methods were performed in accordance with the relevant guidelines and regulations. All clinical tissues used in this study were collected, with the informed consent of the patients, from the First Affliated Hospital of Soochow University (approval no. 2022-284). All animal experiments were carried out in accordance with the Guide for the Care and Use of Experimental Animals Center of Soochow University (approval no. 202306A0850).

## Supplementary information

Supplementary information

Raw materials

## Data Availability

The data presented in this study are all available from the corresponding author upon reasonable request.
