# Supplementary material for: SERBP1 is required for efficient HR repair and cisplatin chemoresistance in lung adenocarcinoma
Source: Cell Death Discov. 2026 Mar 19;12:162. doi: 10.1038/s41420-026-03017-x (PMC13039160; doi:10.1038/s41420-026-03017-x)
Supplement: Supplementary file 1 — Supplementary information [file 41420_2026_3017_MOESM1_ESM.docx]

**Supplementary information**

**SERBP1 is required for efficient HR repair and cisplatin chemoresistance in lung adenocarcinoma**

**
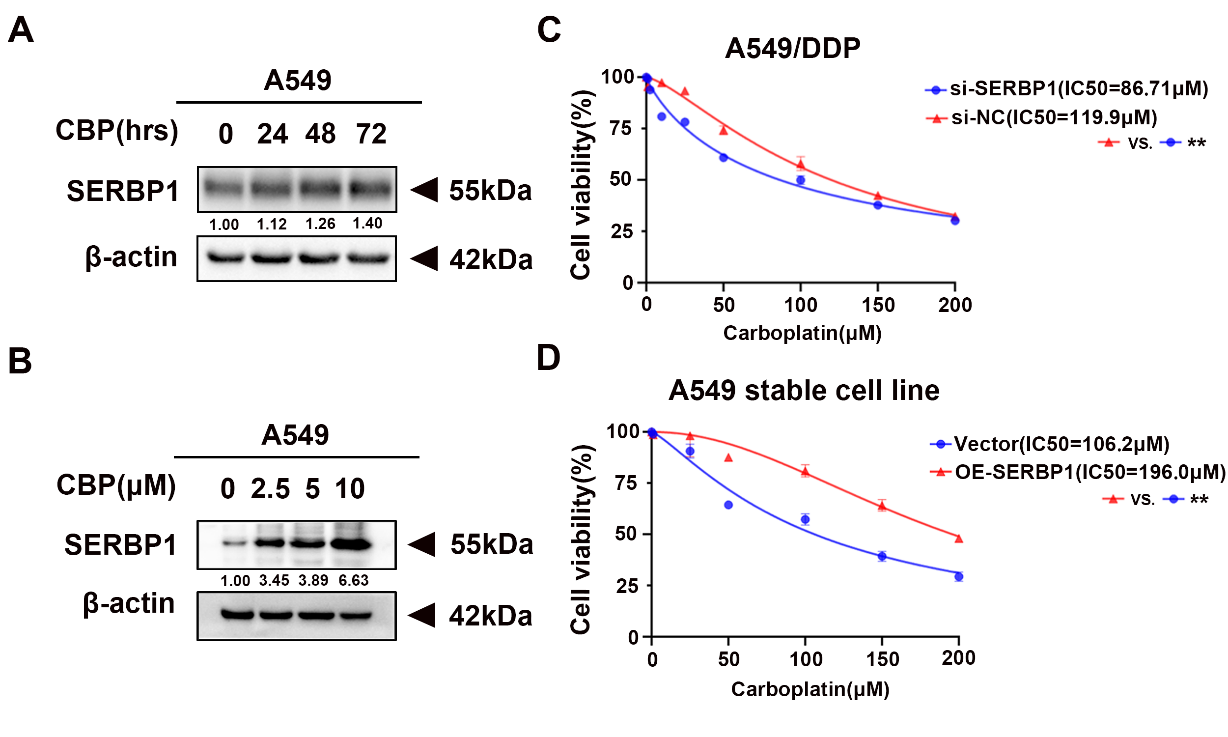
Fig. S1**

**Fig. S1** **SERBP1 promotes carboplatin resistance in LUAD. A** Western blot analysis was used to detect the effect of carboplatin treatment on SERBP1 at different time points in A549 cells. **B** Western blot analysis was used to detect the effect of different concentrations of carboplatin on SERBP1 in A549 cells. **C** The sensitivity of A549/DDP cells transfected with si-SERBP1 or si-NC to 48 h of carboplatin treatment was determined by CCK-8 assays. **D** The sensitivity of SERBP1-overexpressing A549 cells to 48 h of carboplatin treatment was determined via CCK-8 assays. Data are shown as the mean ± SD. ***P* < 0.01.

**Fig. S2**


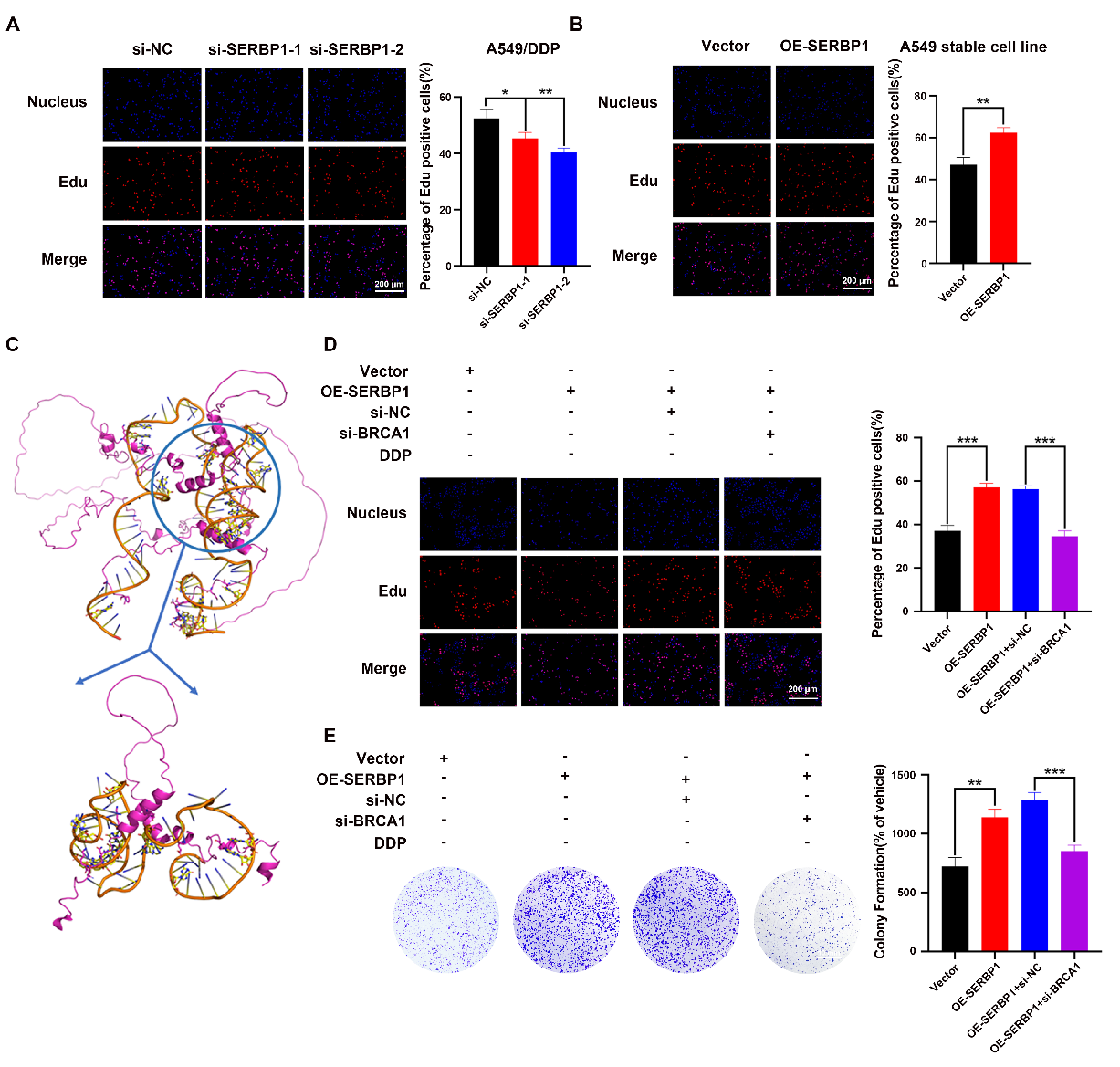


**Fig. S2** **BRCA1 knockdown blocks SERBP1-induced cell proliferation. A** Results of the EdU incorporation assay of si-NC, si-SERBP1-1 and si-SERBP1-2 cells in the absence of cisplatin (5 µM). Scale bar: 200 μm. **B** Results from the EdU assays of vector-transfected and OE-SERBP1 cells in the absence of cisplatin (5 µM). Scale bar: 200 μm. **C** Molecular docking between SERBP1 and BRCA1 mRNA for tertiary structure prediction via NPDock. **D** EdU incorporation assays were performed to assess the proliferation ability of SERBP1-overexpressing cells with BRCA1 knockdown in the absence of cisplatin (5 µM). Scale bar: 200 μm. **E** Colony formation assays of SERBP1-overexpressing cells with BRCA1 knockdown without cisplatin (5 µM). Scale bar: 10 mm. Data are shown as the mean ± SD. **P* < 0.05; ***P* < 0.01; ****P* < 0.001.

**Fig. S3**

**
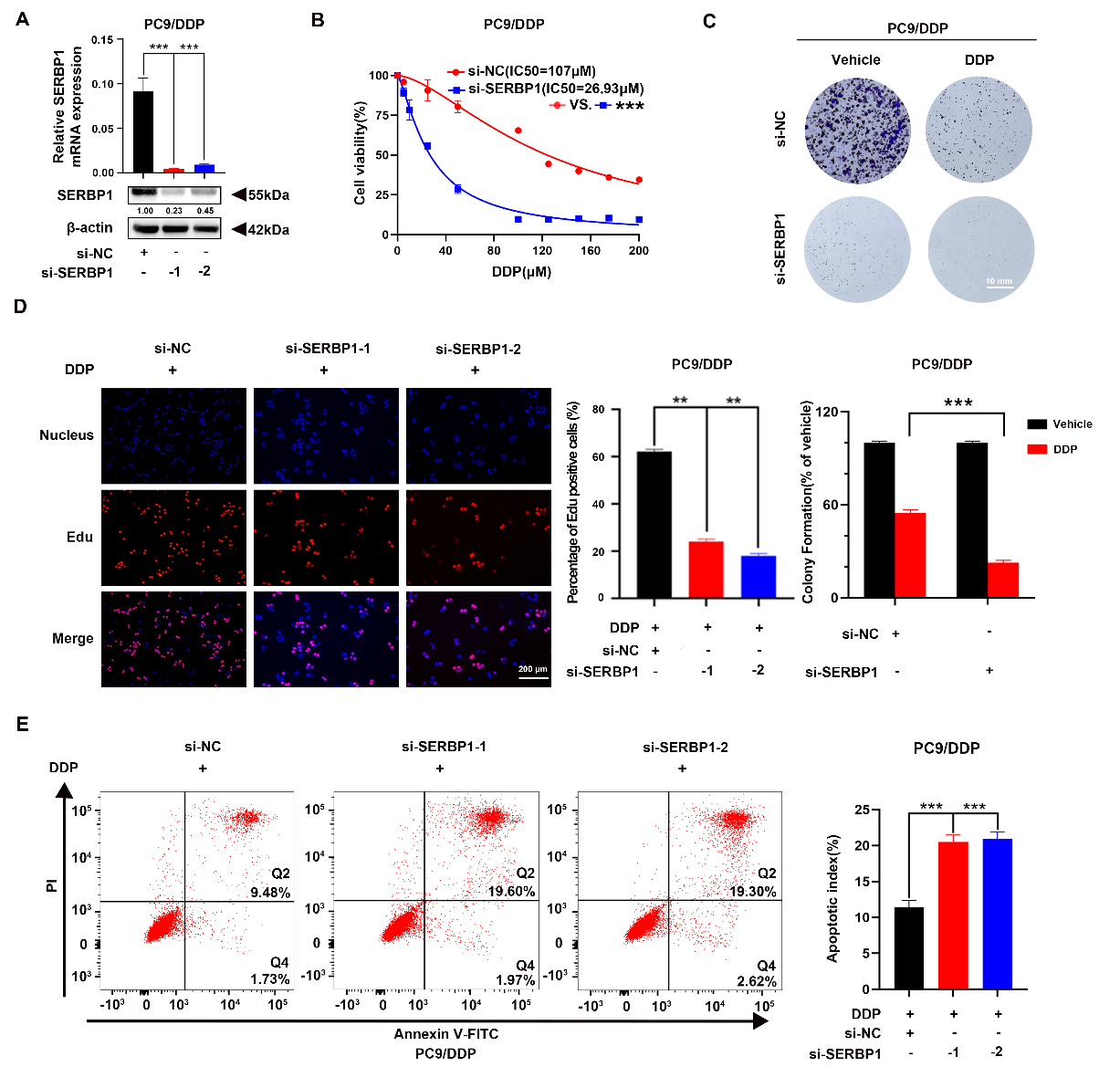
**

**Fig. S3 SERBP1 silencing restores cisplatin sensitivity in PC9/DDP cells. A** SERBP1 mRNA and protein expression was decreased in SERBP1-knockdown PC9/DDP cells. **B** The sensitivity of PC9/DDP cells transfected with si-SERBP1 or si-NC to 48 h of cisplatin treatment was determined via CCK-8 assays. **C** Representative images of the colony formation assay results showing the proliferation of SERBP1-knockdown PC9/DDP cells treated with vehicle (PBS) or 15 μM cisplatin for 48 h; the data are presented below. Scale bar: 10 mm. **D** Results from the EdU assays of si-NC, si-SERBP1-1 and si-SERBP1-2 cells in the presence of cisplatin (15 µM). Scale bar: 200 μm. **E** Flow cytometry assay of the designated cells that were treated with 15 µM cisplatin for 48 h and stained with Annexin V-FITC and PI. The right bar graphs display the statistical analysis results (right panel). Data are shown as the mean ± SD. ***P* < 0.01; ****P* < 0.001.

**Fig. S4**

**
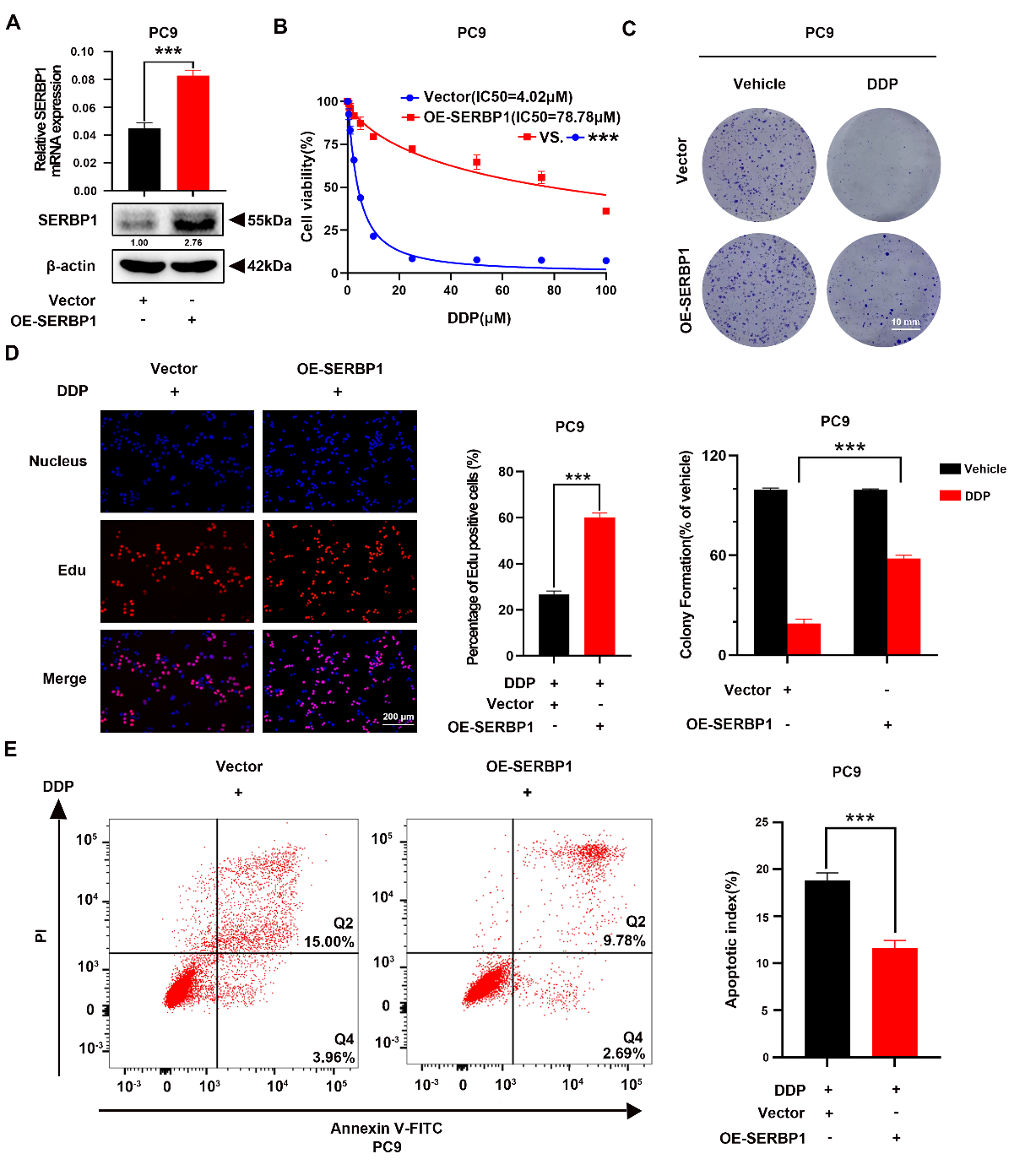
**

**Fig. S4** **SERBP1 overexpression leads to cisplatin resistance in PC9 cells.** **A** SERBP1 mRNA and protein expression was increased in SERBP1-overexpressing PC9 cells. **B** The sensitivity of SERBP1-overexpressing PC9 cells to 48 h of cisplatin treatment was determined by CCK-8 assays. **C** Representative images of the colony formation assay results showing the proliferation of SERBP1-overexpressing PC9 cells treated with vehicle (PBS) or 10 μM cisplatin for 48 h. Scale bar: 10 mm. **D** Results from the EdU incorporation assays of vector-transfected and OE-SERBP1 cells in the presence of cisplatin (10 µM). Scale bar: 200 μm. **E** Flow cytometry assay of the designated cells that were treated with 10 µM cisplatin for 48 h and stained with Annexin V-FITC and PI. The right bar graphs display the statistical analysis (right panel). Data are shown as the mean ± SD. ****P* < 0.001.

**Fig. S5**

**
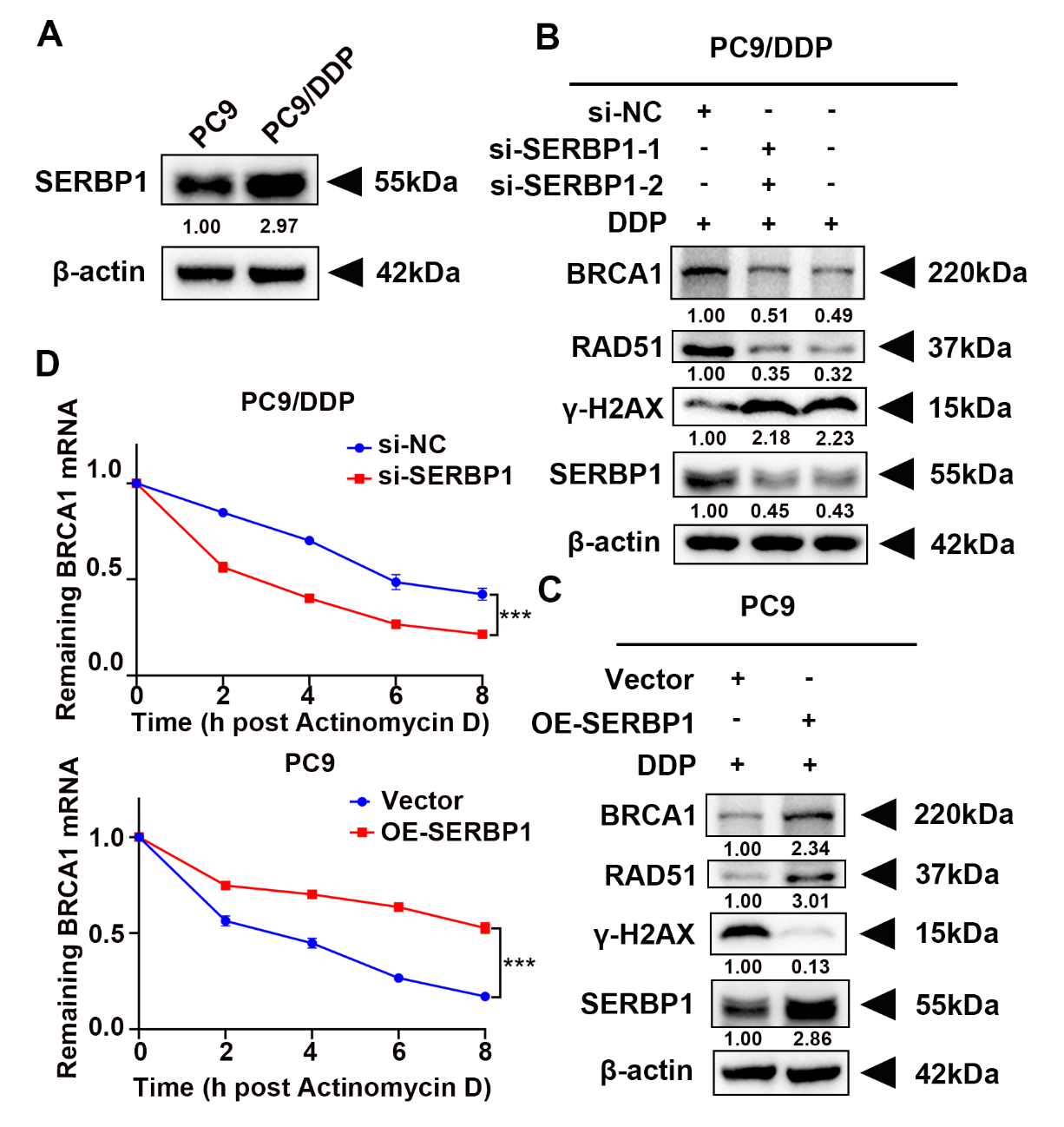
**

**Fig. S5 SERBP1 increases BRCA1 mRNA stability in order to promote cisplatin resistance via HR repair in PC9 cells. A** The expression level of SERBP1 in PC9 and PC9/DDP cells was determined by western blot analysis. **B, C** Western blot analysis was used to verify that SERBP1 regulated HR-related proteins, including BRCA1, RAD51, and γ-H_2_AX in PC9 and PC9/DDP cells. **D** The relative mRNA expression ratio of BRCA1 was analysed by qRT‒PCR in actinomycin D-treated PC9 and PC9/DDP cells at various time points. Data are shown as the mean ± SD. ****P* < 0.001.

**Table S1 Sequences of Primers for Real-time Polymerase Chain Reaction**

| Gene | Forward | Reverse |
| --- | --- | --- |
| SERBP1 | 5'-TAGACCGATTATTGACCGACCT-3' | 5'-GTTTGCCACGAGAATCAAATCC-3' |
| BRCA1 | 5'-GAAACCGTGCCAAAAGACTTC-3' | 5'-CCAAGGTTAGAGAGTTGGACAC-3' |

**Table S2 The Sequences of siRNAs**

| siRNA | Sense | Anti-sense |
| --- | --- | --- |
| Si-NC | 5'-UUCUCCGAACGUGUCACGUTT-3' | 5'-ACGUGACACGUUCGGAGAATT-3' |
| Si-SERBP1-1 | 5'-GCUUAAGAAAGAAGGAAUATT-3' | 5'-UAUUCCUUCUUUCUUAAGCGC-3' |
| Si-SERBP1-2 | 5'-ACUGUCAAAGACGAAUUAATT-3' | 5'-UUAAUUCGUCUUUGACAGUUC-3' |
| Si-SERBP1-3 | 5'-GGGUGAAGGAGGCGAAUUUTT-3' | 5'-AAAUUCGCCUCCUUCACCCTT-3' |
| Si-BRCA1-1 | 5'-GUAUGCAAACAGCUAUAAUTT-3' | 5'-AUUAUAGCUGUUUGCAUACUC-3' |
| Si-BRCA1-2 | 5'-GCAACCUGAGGUCUAUAAATT-3' | 5'-UUUAUAGACCUCAGGUUGCAA-3' |
| Si-BRCA1-3 | 5'-UGAUACUGCUGGGUAUAAUTT-3' | 5'-AUUAUACCCAGCAGUAUCAGU-3' |
