# Supplementary figures and images for: SERBP1 is required for efficient HR repair and cisplatin chemoresistance in lung adenocarcinoma
Source: Cell Death Discov. 2026 Mar 19;12:162. doi: 10.1038/s41420-026-03017-x (PMC13039160; doi:10.1038/s41420-026-03017-x)

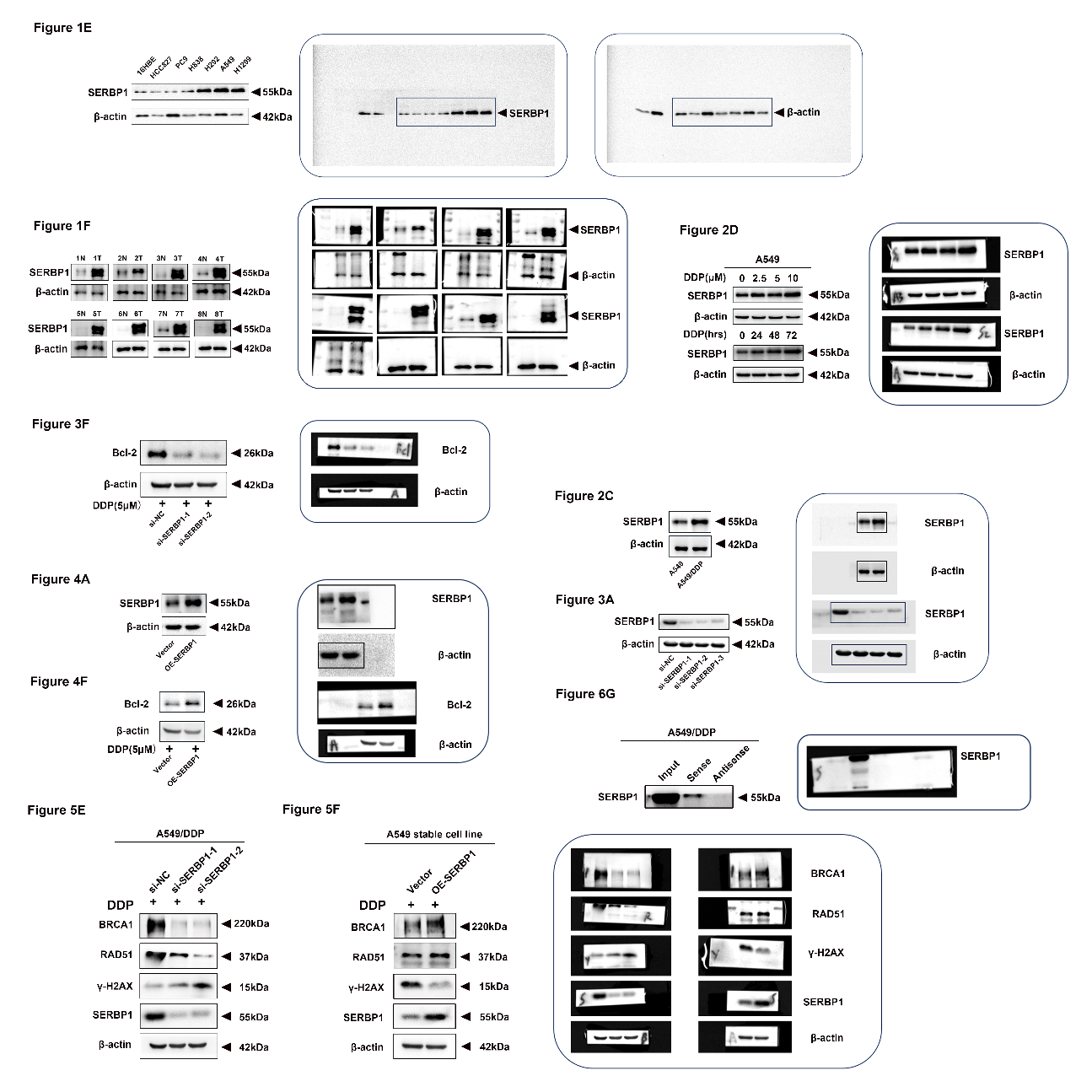


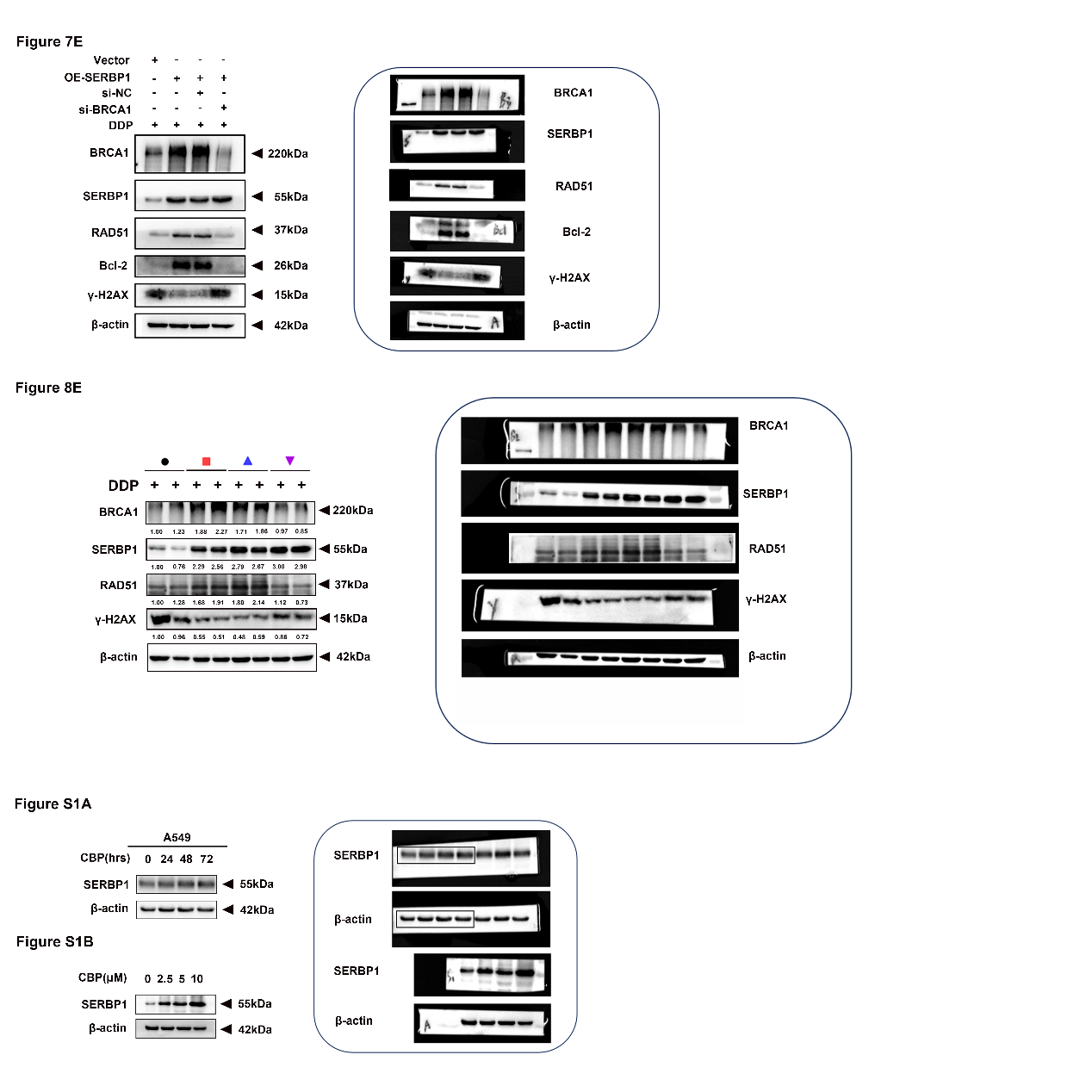

Supplement: Supplementary file 2 — Raw materials [file 41420_2026_3017_MOESM2_ESM.docx]
